# In Memoriam: Rolf Kötter (1961–2010)

**DOI:** 10.1371/journal.pcbi.1000965

**Published:** 2010-10-28

**Authors:** Klaas Enno Stephan, Anthony Randal McIntosh, Claus C. Hilgetag

**Affiliations:** 1 Laboratory for Social and Neural Systems Research, Institute for Empirical Research in Economics, University of Zurich, Zurich, Switzerland; 2 Wellcome Trust Centre for Neuroimaging, Institute of Neurology, University College London, London, United Kingdom; 3 The Rotman Research Institute, Baycrest Center, Toronto, Ontario, Canada; 4 School of Engineering and Science, Jacobs University Bremen, Bremen, Germany; 5 Department of Health Sciences, Boston University, Boston, Massachusetts, United States of America; University of California San Diego, United States of America

**Figure pcbi-1000965-g001:**
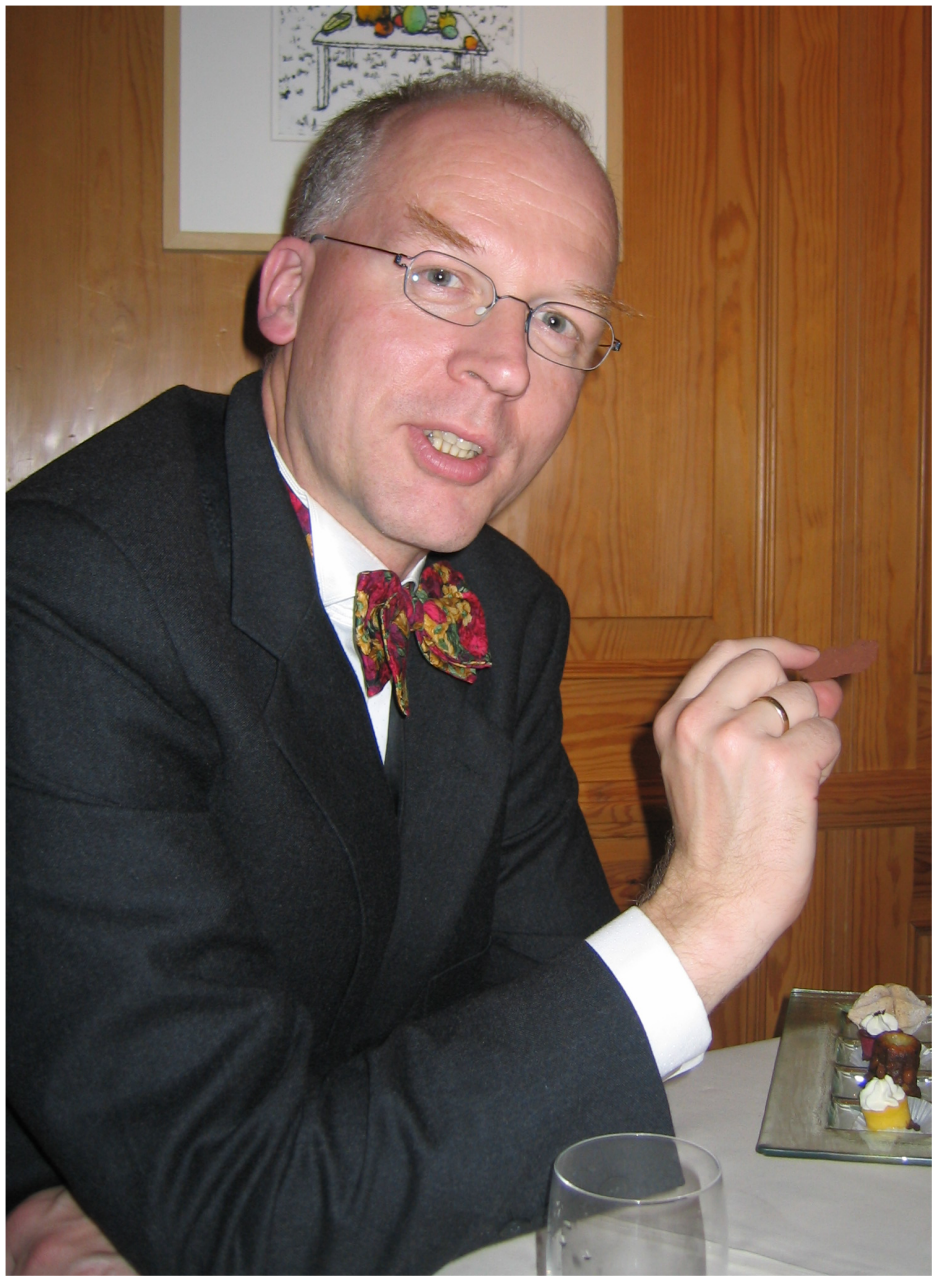
Rolf Kötter.

[Fig pcbi-1000965-g001]Rolf Kötter was *PLoS Computational Biology*'s Reviews Editor for Neuroscience, 2005–2010.

During Rolf Kötter's time as a lecturer at the University of Düsseldorf in Germany, his colleagues were often puzzled by what they perceived as rather incommensurate scientific interests and activities. One was equally likely to find Rolf in his office developing computer models of postsynaptic biochemical signalling cascades [1], or thinking about inter-hemispheric interactions during visuospatial attention [2], in the library writing essays on the history of concepts in neuroscience [3], in his lab in the institute's basement developing novel electrophysiological methods for focal stimulation of brain slices [4], or in the dissection hall teaching gross anatomy to medical students. We believe that these seemingly discordant activities reflected Rolf's appreciation of scientific curiosity per se and his belief that playful exploration (and humour) has an important role in discovering links between supposedly unconnected scientific problems. For example, after reading D'Arcy Thompson's *On Growth and Form*, Rolf amused himself by playing with different geometric transformations of parcellation schemes of the cerebral cortex of the cat. The resulting plots not only served as beautiful, if slightly enigmatic, decorations of his office walls, but also featured in the first and still unique paper on “evolutionary neuro-cartography” [5]. This experience may have helped him to appreciate the importance of novel methods for transforming brain parcellation schemes that were being developed in his group a few years later [6].

Another, perhaps even stronger motivation for Rolf's broad scientific agenda was his conviction that in order to tackle fundamental questions about the brain, it was inevitable that one would have to transcend a single style of thinking, embrace multiple methodologies, and describe mechanisms across multiple levels of neural systems. However, pursuing truly multi-disciplinary work of this sort can be a risky career strategy. While he was highly regarded abroad, he struggled finding a real academic home in his own country, as he had trouble fitting into the traditional disciplines. Eventually, Radboud University Nijmegen in The Netherlands showed foresight in offering him a Chair in Neurophysiology and Neuroinformatics. Rolf started his new job in autumn 2006, full of enthusiasm and excitement about the open-minded new environment and the opportunity of building up a larger research group that would integrate his biological and computational interests. It was only a few months later, in the ascent of his scientific career, that he was diagnosed with a disease that, after a long and courageous battle, eventually proved to be fatal.

Out of Rolf's many achievements, two deserve particular mentioning: the development of the CoCoMac connectivity database and the founding of the Brain Connectivity Workshop series. The origins of the CoCoMac database (Collation of Connectivity data on the Macaque brain) date back to 1996, to a PhD project that was supervised by Rolf and which (initially) dealt with large-scale biophysical models of the macaque cortex. This modelling project required knowledge of the patterns of long-range (inter-areal) connections across the brain, and in the absence of simple rules underlying these patterns, a comprehensive repository of experimental results from anatomical tract tracing studies was needed. This need triggered the construction of the CoCoMac database that, over the following years, superseded the original goals of the PhD project and grew from a simple Excel spreadsheet into a complex relational database system that is publically accessible on the Web (http://www.cocomac.org/). This work not only required many methodological developments [6]–[9], but, more importantly, the firm belief that this database was an important long-term investment. Despite the slow and arduous process to build such a database, Rolf's courage and dedication to the vision of CoCoMac did pay off: over the years, CoCoMac, and associated tools, were central to many of his fundamental papers on brain connectivity, ranging from statistical and graph-theoretical analyses of structural connectivity patterns to large-scale models of brain function (e.g., [10]–[16]). Thus, the CoCoMac project also exemplified the extraordinary combination of idealism and pragmatism that Rolf possessed. While he aspired to ambitious research goals (http://www.hirn.uni-duesseldorf.de/rk/hilbert.htm), he did not shy away from starting with small, imperfect steps as long as they led in the right direction. Beyond his own scientific work, Rolf was keen to ensure that neuroinformatics tools for data analysis and data extraction from CoCoMac were provided to the community, and that interfaces between CoCoMac and other neuroscientific databases were created that would maximise their respective utility [8], [17]. These developments facilitated the use of CoCoMac by research groups all over the world, resulting in numerous seminal publications on organisational principles of brain connectivity and reinforcing the importance of this public database for the neuroscientific community.

Rolf was convinced that neurobiological details and formal computational methods were equally important for tackling fundamental questions about brain function. He was continuously searching for mechanisms by which neurobiological and computational scientists could be brought together so that their respective skills and perspectives would enable projects that neither of them could do alone. In 2001, he was invited to a workshop organised by Randy McIntosh in Toronto (http://www.jsmf.org/meetings/2001/agenda_march_2001.htm). This workshop brought together neuroscientists from various domains, giving each of them just a few minutes and a whiteboard to explain the gist of their scientific questions to colleagues from different disciplines, followed by an intense 30-minute discussion. This format, with its focus on exchange of ideas across traditional boundaries, was exactly what Rolf had been looking for. Inspired by the experience and in partnership with Karl Friston, Rolf organised a workshop on “Functional Brain Connectivity” at Düsseldorf in 2002. This workshop was a tremendous success and resulted in the birth of a community of neuroscientists from different backgrounds working with different methods and at different temporal and spatial scales, but with a shared interest in formal characterisations of neural systems, particularly in terms of connectivity. This multi-disciplinary community became Rolf's intellectual home. He loved the intense discussions at the annual meetings of the “Brain Connectivity Workshop” or BCW, as the meeting eventually became known, and he cherished the constructive and collaborative atmosphere amongst its attendants who faithfully reconvened from year to year in ever greater numbers (http://www.hirnforschung.net/bcw). In the last stage of his illness, he could only witness the most recent BCW (2010, at Berlin) from a distance, but he was eager to hear about every little detail of the meeting.

Rolf was a wonderful mentor to his students, always accessible, and never failing to take them and their ideas seriously. Amongst his colleagues, he was highly respected for his collegiality, loyalty, and honesty, making him both a tremendous collaborator and friend. At the time of ever increasing specialisation in modern science, he was one of the few remaining Renaissance men, with an impressively broad range of knowledge and skills, genuine idealism, and a far-reaching vision. He will be missed dearly, but his impact on systems neuroscience will continue to be felt.
